# Anuria Following Solitary Left Renal Vein Ligation: An Old Surgery Myth Is Rigorously Challenged

**DOI:** 10.7759/cureus.97430

**Published:** 2025-11-21

**Authors:** Macaulay A Onuigbo, Oni Balonfentse

**Affiliations:** 1 Medicine, The Robert Larner, M.D. College of Medicine, University of Vermont, Burlington, USA

**Keywords:** acute kidney injury, anuria, end-stage renal disease (esrd), left renal vein ligation, solitary functional kidney, solitary kidney

## Abstract

A consensus was established over the past several decades that left renal vein ligation, which is sometimes performed intraoperatively to improve surgical exposure and to control bleeding, was an innocuous and safe procedure. This surgical myth is primarily based on collateral venous pathways draining the left kidney post-ligation, with outcomes ranging from mild reversible transient renal dysfunction to no impact on renal function. Nevertheless, there have been some reports of transient renal failure and, rarely, irreversible end-stage renal disease following this procedure. We recently encountered post-operative anuria on post-operative day one with rapidly escalating serum creatinine that tripled in 24 hours and continued to rise. The patient soon started intermittent hemodialysis on post-operative day two and required a total of three alternate daily hemodialysis sessions before urine output returned. Hemodialysis was discontinued, and serum creatinine continued to decrease spontaneously thereafter. She was discharged home on post-operative day eight. Clearly, left renal vein ligation is not always a safe and innocuous procedure, more so in a patient with a single functioning left kidney. This is a call for increased caution and to limit this procedure or, otherwise, to consider vascular surgery repair and re-anastomosis of the ligated left renal vein, before wound closure.

## Introduction

Experience and consensus had been built around the concept that elective left renal vein ligation, which is sometimes performed to improve surgical exposure and to control bleeding, was an innocuous and safe procedure. This surgical myth is primarily based on collateral venous pathways draining the left kidney post ligation, with outcomes ranging from mild reversible transient renal dysfunction to no impact on renal function [1,2]. Nevertheless, there have been some reports of transient renal failure, sometimes requiring temporary hemodialysis, and, rarely, irreversible end-stage renal disease following left renal vein ligation [3-6]. The surgical literature supporting this myth involved patients with two functioning kidneys, and often, with prior inferior vena cava obstruction and therefore with a priori well-developed left renal vein collaterals [1,2]. Clearly, such considerations simply do not apply to solitary functioning left kidneys without preexisting inferior vena cava obstruction [3-6]. We recently encountered post-operative anuria in a solitary left kidney following an elective left renal vein ligation during an open retroperitoneal peri-aortic lymph node biopsy procedure. This experience was a clear manifestation of the significant risks and complications that could follow this surgical intervention. However, it must be acknowledged that some other observations have led other investigators to conclude that, even with the sudden obstruction to venous outflow that occurs after an elective left renal vein ligation, the left kidney can still promptly develop an adequate network of collateral venous drainage capable of supporting normal renal function [7]. Indeed, other investigators have argued that the restoration of left renal vein continuity after left renal vein division and ligation may be unnecessary since renal compromise and hematuria were not encountered in their long-term analysis of 56 patients seen between 1992 and 2007 in Florida [1]. Another report from 2009 concluded that selective left renal vein ligation and division during aortic occlusive surgery did not compromise the left kidney [8].

## Case presentation

**Figure 1:**
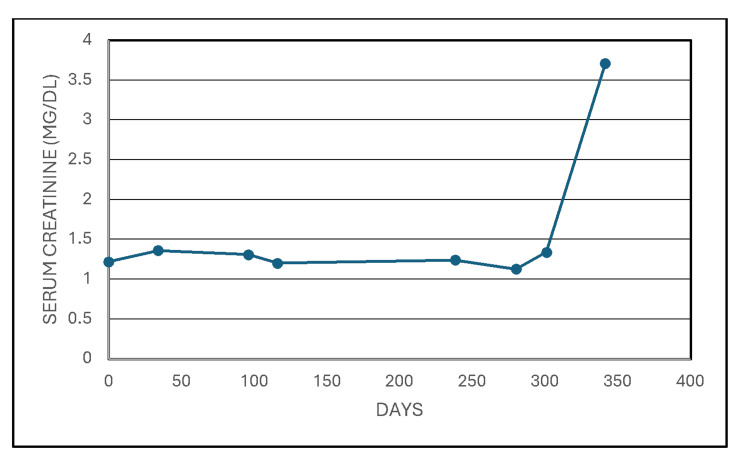
Despite stable vital signs, by post-operative day one, serum creatinine had almost tripled to 3.71 mg/dL.

A 55-year-old woman with type 2 diabetes mellitus, hypertension, prior multiple non-diagnostic imaging-guided needle aspiration biopsies of a periaortic lymph node, past robot-assisted laparoscopic right radical nephroureterectomy for urothelial carcinoma in October 2020, successfully underwent an open retroperitoneal lymph node biopsy with left renal vein ligation. Estimated blood loss was 100 mL, and urine output during the surgery was 125 mL. No complications were reported during the procedure, and the patient tolerated the procedure well. Post operatively, she was in a hemodynamically stable condition. She had a Foley catheter in place until ambulatory, and she quickly transitioned to a clear liquid diet. Preoperative creatinine was 1.3 mg/dL. Despite stable vital signs, by post-operative day (POD) one, serum creatinine had almost tripled to 3.71 mg/dL (Figure 1).

**Figure 2:**
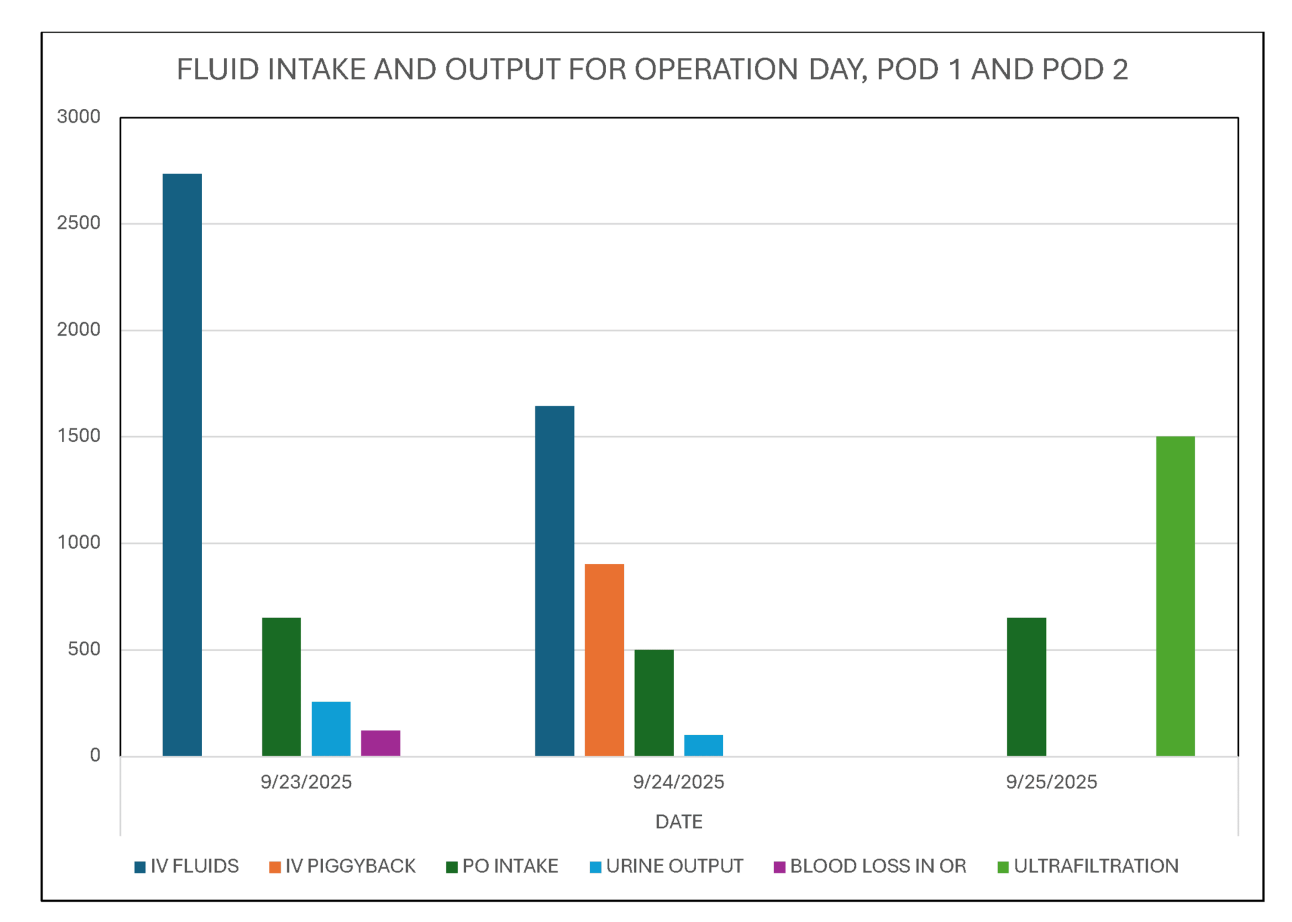
She was anuric the first morning following the procedure.

She was anuric the first morning following the procedure, and a urine bladder scan that morning on POD 1 revealed zero urine, and the Foley catheter was empty (Figure 2).

**Figure 3:**
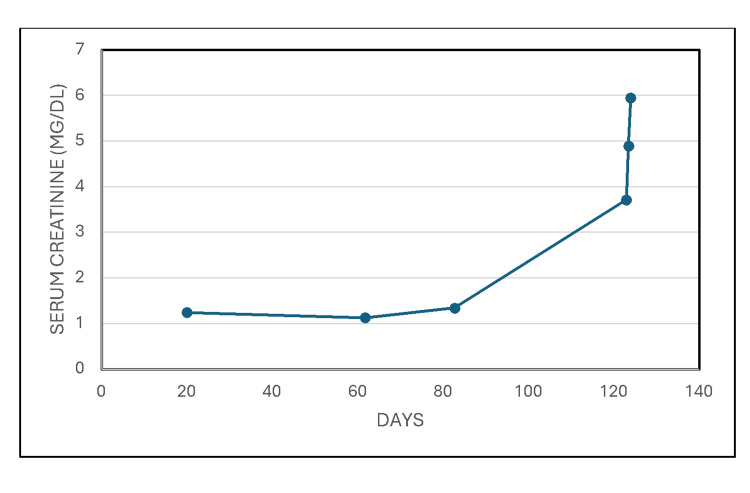
Serum creatinine subsequently continued to rise, with associated hyperphosphatemia.

Serum creatinine subsequently continued to rise, with associated hyperphosphatemia (Figure 3).

**Figure 4:**
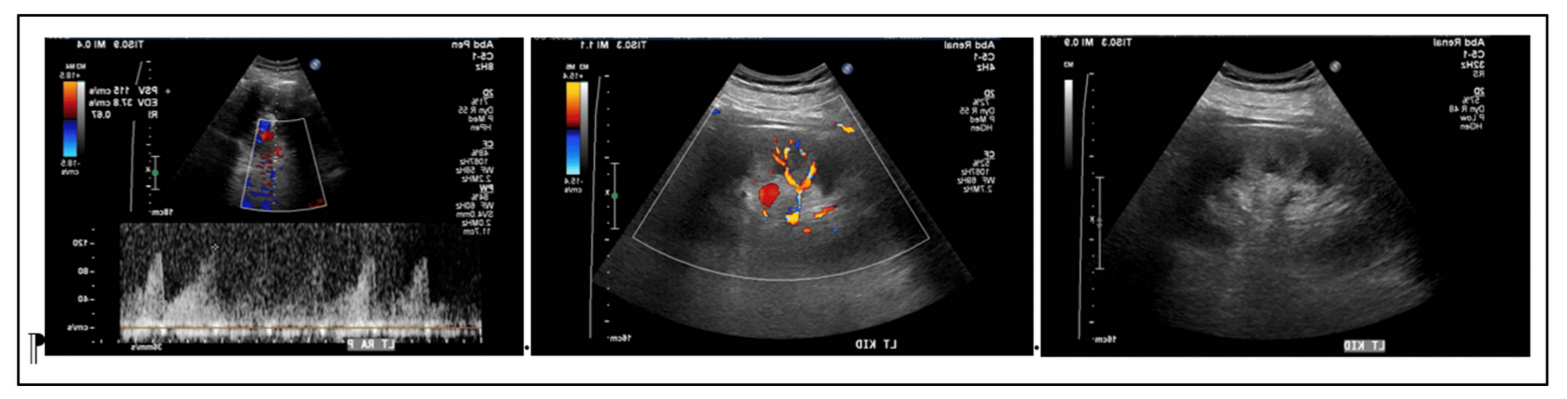
Left kidney ultrasound examination and left renal artery and vein duplex examination on post-operative day one.

Physical examination was normal, except for post-operative abdominal status. She had no peripheral edema. With persistent anuria and rapidly increasing creatinine, a tunneled hemodialysis catheter was promptly placed later in the afternoon of POD 1. She had her first hemodialysis treatment on POD 2. A renal ultrasound with renal artery and vein Duplex on POD 1 revealed that the left kidney measured 15 cm, and duplex exam of the left kidney vasculature showed no evidence for renal artery stenosis (Figure 4). The proximal left renal artery peak systolic velocity, which, in early 2022, was 275 cm/s, was now significantly reduced to 115 cm/s. The left renal vein was visualized distally and was widely patent (Figure 4).

**Figure 5:**
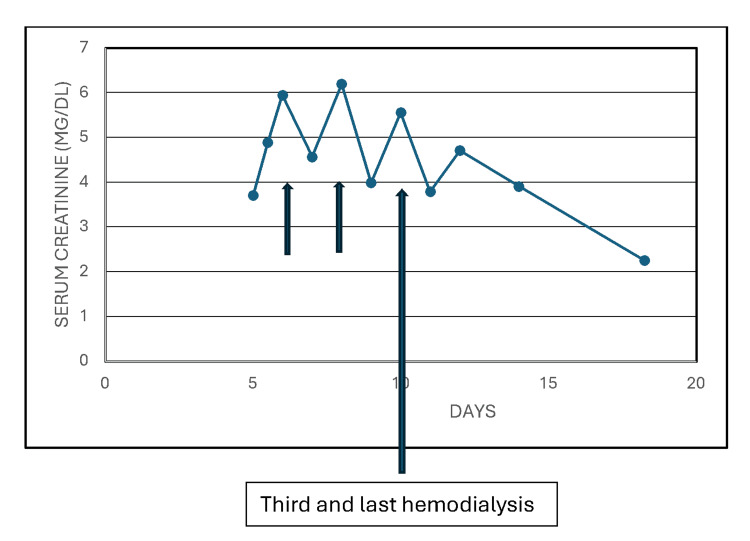
She received three alternate daily hemodialysis treatments for persistent oligoanuria and serum creatinine peaked at 6.2 mg/dL on postoperative day (POD) 4.

By POD 3, she again started making clear yellow urine with SG of 1.014, pH of 5.5, 2+ blood, 3+ proteinuria, few WBC, few RBC, few casts, no bacteria, and a few renal epithelial cells. She received alternate daily hemodialysis treatments for persistent oligoanuria. Serum creatinine peaked at 6.2 mg/dL on POD 4 (Figure 5). The third hemodialysis treatment was on POD 6, and she subsequently made more urine, and hemodialysis was discontinued. She was discharged home on POD 8. Serum creatinine continued to decrease spontaneously, off hemodialysis, and had decreased to 2.26 mg/dL by POD 13 (Figure 5). Hyperphosphatemia was normalized by POD 10.

## Discussion

Case reports, case series, and retrospective reviews have described variable outcomes following left renal vein ligation [1-8]. Some patients hardly experienced any significant change in renal function, others quickly recovered renal function through collateral venous drainage, and others progressed to irreversible renal failure [1-8]. Even in patients with two kidneys, some reports have demonstrated an increased risk of postoperative azotemia. In one review of 332 abdominal aortic aneurysm repairs, nine of 13 patients with left renal vein ligation developed significant creatinine rises compared to 21 of 319 without ligation [9]. In solitary kidney patients, the absence of collateral outflow makes ligation particularly dangerous, and irreversible renal loss and need for permanent dialysis have been described [4]. Recent studies continue to highlight the variability of renal tolerance and emphasize cautious, individualized operative planning regarding the practice of pre-emptive left renal vein ligation performed intraoperatively to improve surgical exposure and to control bleeding. Thus, some authors have argued that the ligation of the left renal vein increases the risk of post-operative renal complications and that its use should be selective [9]. It is arguable that the impact of increased venous hypertension following left renal vein ligation is comparable to congestive nephropathy from inferior vena cava hypertension secondary to worsening right heart failure, one of the components of cardiorenal syndrome as classically described by Ross [10]. Consequently, some other investigators insist that vascular surgery repair and re-anastomosis of the ligated left renal vein were indeed mandatory and necessary to mitigate renal injury [11-14].

## Conclusions

For the very first time, we have demonstrated in our patient the finding that left renal arterial blood flow was significantly compromised within the first 24 hours of the left renal vein ligation. Proximal left renal artery peak systolic velocity, which, in early 2022, was 275 cm/s, was significantly reduced to 115 cm/s. The observation that the single kidney after about 36 hours had started making clear yellow urine again, albeit with some abnormalities on urinalysis, was quite an impressive and fascinating observation. These observations raise exciting and interesting questions regarding our current state of understanding of normal renal physiology with regard to renal perfusion and renal artery and renal vein modalities. Furthermore, these findings, as stated above, raise exciting and parallel déjà vu similarities with the concept of congestive nephropathy from heart failure associated with inferior vena cava hypertension. Post-operative anuria following the ligation of the left renal vein in our patient, followed by renewed urine production, would be explained on the basis of initial insurmountable venous hypertension shutting down the solitary left kidney function, and then progressive “re-opening” of the venous collaterals and the re-establishment over the ensuing days, and may be weeks, of sufficient left renal collateral vein drainage to hopefully normalize her kidney function.

Thus, we ask the question "How safe is left renal vein ligation in an adult patient with a lone functioning left kidney?" The controversy over the safety of this procedure continues. Despite the ongoing controversies regarding this procedure, from our experience, we must vigorously re-echo the position of Miedema and Stubenbord in 1984, that the ligation of the left renal vein is not safe! It should be avoided where possible. Nevertheless, if it must be done, immediate intra-operative vascular surgical repair and re-anastomosis of the ligated and divided left renal vein should be considered as mandatory. Indeed, a recent 2025 report from Korea has lent further strong support to the concept of left renal vein transection and reconstruction for renal preservation.
